# Investigating the mediating influence of distress tolerance on the relationship between existential thinking, sense of coherence, and the severity of mourning among families who lost a loved one to COVID‐19: A cross‐sectional study

**DOI:** 10.1002/hsr2.1518

**Published:** 2023-08-31

**Authors:** Soheila Khalafi kasalani, Mina Karami, Shahriar Dargahi

**Affiliations:** ^1^ Department of Psychology Islamic Azad University Khalkhal Tehran Iran; ^2^ Department of Medical Sciences Islamic Azad University Tehran Iran; ^3^ Department of Family Health, Social Determinants of Health Research Center Ardabil University of Medical Sciences Ardabil Iran

**Keywords:** covid‐19, distress tolerance, existential thinking, sense of coherence, severity of mourning

## Abstract

**Background and Aims:**

The objective of the current study was to examine how distress tolerance functions as a mediator in the relationship between existential thinking, sense of coherence, and the severity of mourning experienced by families who have lost a loved one to COVID‐19.

**Methods:**

The present study employed a descriptive correlational research design, targeting family members of those who passed away due to COVID‐19 in the city of Mianeh in 2022. A sample of 160 individuals was selected for statistical analysis. The research instruments used in this study consisted of Flensberg's sense of coherence questionnaire (2006), Simmons and Gaher's emotional distress tolerance questionnaire (2005), Sugbart and Scott's grief experience questionnaire (1989), and Branton Scherer's existential thinking questionnaire (2006). The collected data were analyzed using path analysis, as well as SPSS and Amos software.

**Results:**

The findings of the study revealed a significant correlation coefficient between existential thinking (*r* = 0.465), sense of coherence (*r* = 0.401), and distress tolerance (*r* = 0.521) with the severity of mourning experienced by families who lost a loved one to COVID‐19. Moreover, the results indicated a positive and significant relationship (*p* > 0.01) between distress tolerance and sense of coherence (*r* = 0.126), as well as between distress tolerance and existential thinking (*r* = 0.059) among the bereaved families. However, the bootstrap test results suggested that distress tolerance did not mediate the relationship between sense of coherence and the severity of mourning in the families of COVID‐19 victims.

**Conclusion:**

Consistent with prior research, the current study's findings indicated that both existential thinking and sense of coherence had a direct impact on the severity of mourning experienced by families who lost a loved one to COVID‐19. Additionally, the results revealed that the influence of existential thinking on the severity of mourning was mediated indirectly by increasing distress tolerance.

## Key points

- The severity of grief among families who lost a loved one due to Covid‐19 can be affected by the psychological factors of different people.
- Existential thinking had a direct and indirect effect on the severity of grief, so existential psychotherapy can be a good help to increase psychological capacity and accelerate the process of grief treatment.

## 1 INTRODUCTION

One of the most significant challenges for families who have lost a loved one to COVID‐19 is dealing with the burial process and the inability to hold traditional mourning ceremonies. Due to the nature of the virus, special health conditions must be met for the burial of COVID‐19 victims, and families are not permitted to view the body or say their goodbyes. These circumstances can lead to abnormal or complicated grief experiences for family members.^1^ Additionally, the degree of dependence that each individual had on the deceased can also hinder the natural mourning process. Abnormal grief reactions may manifest as disruptions in the family's normal functioning, avoidance of discussing death‐related topics, and severe grief symptoms.^2^ Survivors' emotions may vary based on the sense of loss around those who passed away. For instance, some may feel guilty or self‐blame due to the isolation, quarantine, and special hospitalization conditions for their loved ones, as well as the inability to care for them during their illness.^3^ All of these factors can contribute to the severity of mourning experienced by individuals during an epidemic. Therefore, how individuals perceive and process the loss of their loved ones can be influenced by various factors after their passing.

In situations of distress, one of the key factors that individuals need to possess is the ability to endure such distress. Distress tolerance refers to a person's capacity to withstand and endure negative emotional states.^4^ Distress tolerance is a complex construct that encompasses multiple dimensions, such as the ability to bear negative emotions, the capacity to evaluate and accept one's emotional state, and the methods of emotion regulation that the individual employs.^5^ Some studies suggest that distress tolerance is one of the psychological factors that contribute to psychological vulnerability,^6^ and can play a mediating role in the complex grief and posttraumatic stress experienced by bereaved families.^7^ The level of distress tolerance among bereaved individuals can be influenced by various psychological factors, including sense of coherence, which is considered to be an important variable in this context. Sense of coherence refers to the sense of meaning, control, and understandability that individuals experience in their lives, and it can have a significant impact on their mental health.^8^ Therefore, helping bereaved individuals maintain their sense of coherence is crucial. Research findings by Bolen and Connor suggest that creating meaning with a sense of coherence can increase positive responses among bereaved individuals^9^ and aid in regulating their emotions.^8^ Sense of coherence can act as a critical source of psychological resilience and has been linked to positive outcomes such as hope, psychological well‐being, positive affect, and life satisfaction.^10^

The structure of spiritual and existential thinking is considered to be one of the primary protectors of mental health during crises. Existential thinking refers to an individual's desire to contemplate fundamental life issues, such as the meaning and purpose of life, death, emptiness, and alienation.^11^ Researchers suggest that exploring these existential issues may help individuals endure distress by increasing their awareness of existential issues and emphasizing the importance of discussing and reflecting on these issues.^12^ Furthermore, the existential attitude can stimulate healthy behaviors by encouraging individuals to search for the meaning of life and suffering.^13^ Studies have also demonstrated that existential thinking plays a mediating role in the relationship between death anxiety and empathy with COVID‐19 patients^14^ and can enhance the quality of working life for those involved in caring for dying patients.^15^

The lifestyle changes and intense grieving experienced by individuals who have lost loved ones due to COVID‐19 have led to an increase in mental health‐related problems among the family members of the deceased, which is a cause for concern. It is important to protect these survivors from the potential hardships associated with losing a loved one. However, possessing certain psychological capabilities can help reduce the duration and intensity of the grieving process, or mitigate its negative impact on an individual's life. Despite this, few studies have explored the psychological mechanisms that affect the bereavement experienced by families of COVID‐19 victims, suggesting that the importance of this issue has been understudied in the field of mental health. Therefore, there is a need for research that can shed light on the psychological factors influencing the grieving process among these families. In light of this gap in the literature, the present study was conducted to investigate the mediating role of distress tolerance in the relationship between existential thinking, sense of coherence, and the severity of mourning experienced by families who have lost a loved one to COVID‐19.

## 2 MATERIALS AND METHODS

The present study was a descriptive correlational study conducted intermittently starting from the beginning of 2022 in the city of Tabriz. The study population consisted of family members of individuals who had died due to COVID‐19 in Tabriz, including spouses, parents, children, and siblings of the deceased. A purposive sampling method was employed, whereby one of the main family members of the deceased, including the spouse, child over 18 years old, father, mother, sister, or brother, was contacted and informed about the research objectives and the confidentiality of the results. A total of 160 participants completed the questionnaires in person.

To be eligible for the study, participants had to meet certain criteria, which included: the deceased must have died with a confirmed diagnosis of COVID‐19 in 2022 or later; the participant must not have sought psychological help for a psychiatric disorder in the year before the incident; the participant must have had a first‐degree blood relationship with the deceased; and the participant must have provided consent to participate and complete the questionnaire.

### 2.1 Distress tolerance questionnaire

Simmons and Gaher developed a self‐report index consisting of 15 items and four subscales, which include emotional distress tolerance, absorption of negative emotions, estimation of mental distress, and adjustment of efforts to relieve distress. Respondents rate each item using a five‐point Likert scale ranging from 1 (indicating complete agreement with the desired option) to 5 (indicating complete disagreement with the desired option). Higher scores on the scale suggest greater distress tolerance. The *⍺* coefficients for the subscales were 0.72, 0.82, 0.78, and 0.70, respectively, and 0.82 for the whole scale. After 6 months, the intra‐class correlation was found to be 0.16. The scale also demonstrated good criterion validity and initial convergence, as indicated by previous research.^5^ In local research, Cronbach's *⍺* coefficient for the whole scale was 0.93, and the coefficient obtained through the retest method was 0.61.^16^

### 2.2 Existential thinking questionnaire

Branton Scherer developed the existential thinking questionnaire in 2006 to measure existential intelligence. The questionnaire uses a 6‐point Likert scale (always, almost always, often, sometimes, never, and I don't know) to assess subjects' engagement with existential concepts. The questionnaire consists of 11 items and a general subscale, and the total score ranges from 11 to 55, with higher scores indicating a greater degree of preoccupation with existential concepts. The original version of the questionnaire exhibited high reliability, with an internal consistency of 0.93. The questionnaire's single‐factor structure has been supported by numerous studies.^17^ A local study found a Cronbach's *⍺* coefficient of 0.88 and a test−retest correlation coefficient of 0.75, indicating the questionnaire's good reliability.^11^

### 2.3 Grief experience questionnaire

The grief experience questionnaire, first introduced by Barrett and Scott, consists of 34 questions designed to evaluate several aspects of bereavement. This questionnaire applies to various types of loss and death. The factors assessed include physical reactions, general grief reaction, search for an explanation, loss of support, labeling, guilt, responsibility, shame, rejection, self‐destructive behavior, and unique reactions. In the original study by Barrett and Scott, the internal consistency of the questionnaire was found to be high, with a Cronbach's *⍺* coefficient of 0.97. Cronbach's *⍺* coefficients for the 11 factors were as follows: physical reactions (0.79), general grief reactions (0.68), trying to find an explanation (0.68), loss of support (0.86), being labeled (0.88), guilt (0.89), responsibility (0.88), shame (0.83), rejection (0.87), self‐destructive behavior (0.79), and unique reactions (0.79).^18^

### 2.4 Antonevsky's sense of coherence

Antonevsky developed the abbreviated version of sense of coherence in 1987,^19^ which comprises 13 items measuring three dimensions: comprehensibility (5 items), manageability (4 items), and meaningfulness (4 items). The scale uses a 5‐point Likert scale, and three items are scored in reverse. Scores on this scale range from 14 to 4, with higher scores indicating greater coherence. The scale provides not only separate scores for each subscale but also an overall score. In Iran, Mohammadzadeh et al. standardized this questionnaire for Iranian students after translating it. Cronbach's *⍺* values for male and female students were 0.75 and 0.78, respectively, indicating acceptable internal consistency. The concurrent validity of this scale with the 45‐item mental toughness questionnaire was 0.54. The overall scale demonstrated good test−retest reliability with a coefficient of 0.66. To assess the validity of the questionnaire, the researchers examined the relationship between the understanding, management, and meaningfulness subscales and the total score of the questionnaire. The results indicated correlations of 0.86, 0.81, and 0.76, respectively, indicating that the scale is valid and reliable.^20^

## 3 FINDINGS

Table 1 displays the age distribution of individuals who have died from Covid‐19.

**Table 1:** Age distribution of the individuals who died from Covid‐19.

Age	Frequency	Percentage
<20	3	1.8
21−40	15	9.6
41−60	45	28.8
61−80	93	57.7
>81	4	2.1
Total	160	100

Based on the results obtained in Table 1, it is evident that the largest number of respondents falls within the age range of 61−80 years, while the smallest number is in the under‐20 age group.

Table 2 shows the frequency of the relationship between the participants with the deceased person caused by the Covid‐19. That among them is the most type of relationship of children's share.

**Table 2:** The type of relationship between the participant and the person who died from Covid‐19.

Relationship	Frequency	Percentage
Spouse	37	23
Children	69	43
Siblings	27	17
Parents	27	17

Table 3 shows descriptive statistics (mean, standard deviation, maximum, and minimum) of research variables. According to the table of mean and standard deviation of distress tolerance is (60.44 ± 3.10), existential thinking (38.78 ± 3.32), sense of coherence (111.31 ± 10.00), and severity of mourning (110.7 ± 10.26).

**Table 3:** Descriptive statistics.

Component	Minimum	Maximum	Mean	Standard deviation
Distress tolerance	67	54	60.44	3.10
Existential thinking	47	32	38.78	3.32
Sense of coherence	158	86	111.31	10.00
Severity of mourning	156	89	110.7	10.26

As shown in Table 4, there exists a significant correlation between existential thinking and distress tolerance (*r* = 0.059) and the severity of mourning (*r* = 0.465) (*p* < 0.01). Furthermore, sense of coherence demonstrates a positive and significant correlation with distress tolerance (*r* = 0.126) and the severity of mourning (*r* = 0.401). Additionally, distress tolerance exhibits a positive and significant relationship with the severity of mourning (*r* = 0.521).

**Table 4:** Correlation matrix of the variables.

	1	2	3	4
Sense of coherence	1			
Existential thinking	0.213**	1		
Distress tolerance	0.126*	0.059**	1	
Severity of mourning	0.401**	0.465**	0.521**	1

* *p* > 0.05

** *p* > 0.01.

As shown in Table 5, the value of the *χ* ^2^ index is not significant (*p* > 0.050), and all fit indices of the model have also reached the reasonable fit criterion. Goodness of fit index and comparative fit index are above 0.90, which are reasonable for model fitness. The root mean square error of approximation was also 0.056 which is reasonable.

**Table 5:** Model fit indices.

Index	*χ* ^2^	*p*	GFI	AGFI	CFI	RMSEA
Value	1.905	0.206	0.991	0.958	0.991	0.056

Abbreviations: AGFI, adjusted goodness‐of‐fit index; CFI, comparative fit index; GFI, goodness‐of‐fit index; RMSEA, root mean square error of approximation.

According to Table 6 and the paths of the model, it can be observed that sense of coherence has a significant positive direct effect on the severity of mourning among the families of the deceased from COVID‐19 (*β* = 0.480), but it does not significantly impact distress tolerance (*β* = 0.007). Furthermore, existential thinking has a significant negative direct effect on the severity of mourning (*β* = −0.297) and a significant positive direct effect on distress tolerance (*β* = 0.152). Additionally, the severity of mourning has a significant negative direct effect on distress tolerance (*β* = −0.372).

**Table 6:** Standard and non‐standard coefficients in the proposed path model.

Variables	*B*	*β*	SE	T (CR)	*p*
A sense of coherence → distress tolerance	−0.006	−0.007	0.047	−0.134	0.894
A sense of coherence → severity of mourning	0.252	0.480	0.085	2.984	0.006
Existential thinking → distress tolerance	0.276	0.152	0.023	12.171	0.001
Existential thinking → severity of mourning	−0.309	−0.297	0.041	−7.463	0.001
Distress tolerance → severity of mourning	−0.234	−0.372	0.025	−9.353	0.001

**Figure 1:**
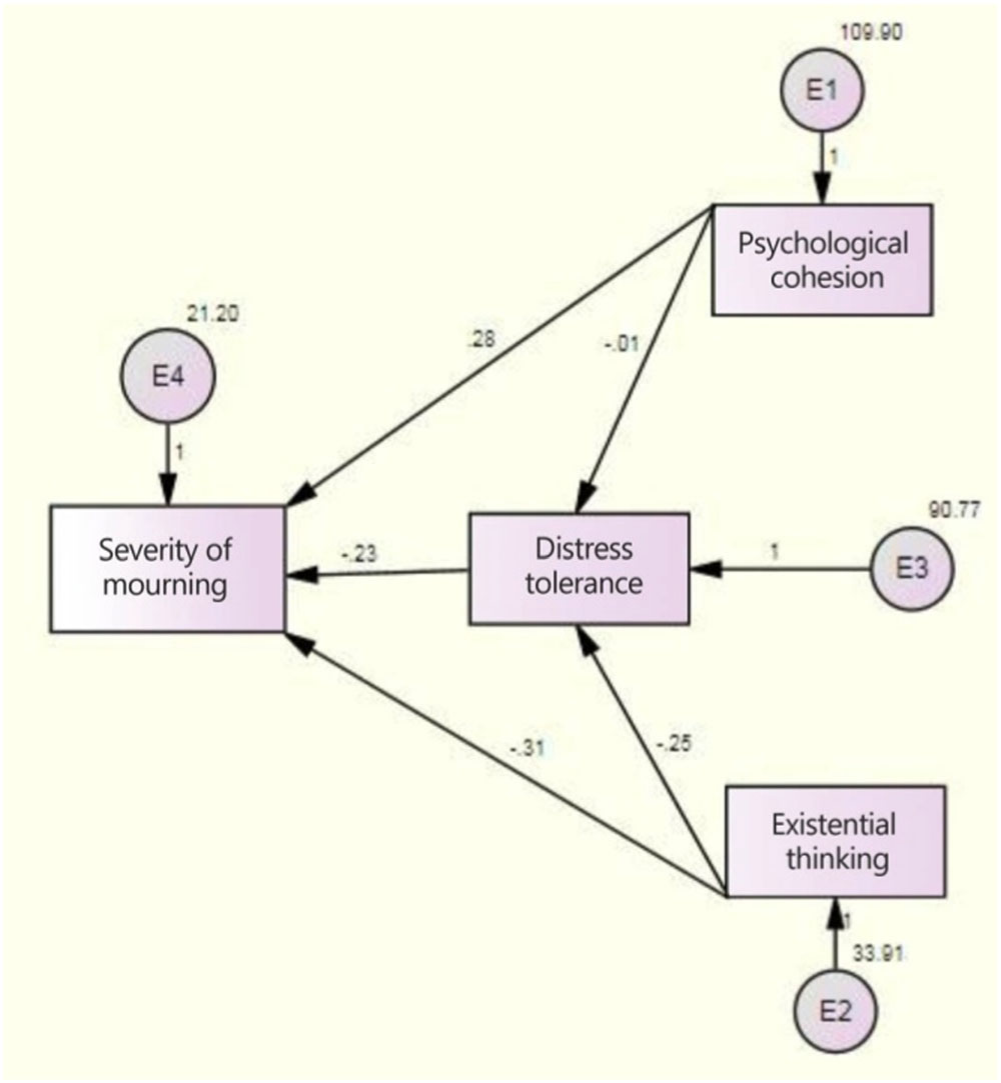
The model for predicting the severity of mourning (standard mode). Figure 1 shows the direct and indirect paths in the standardized model.

**Figure 2:**
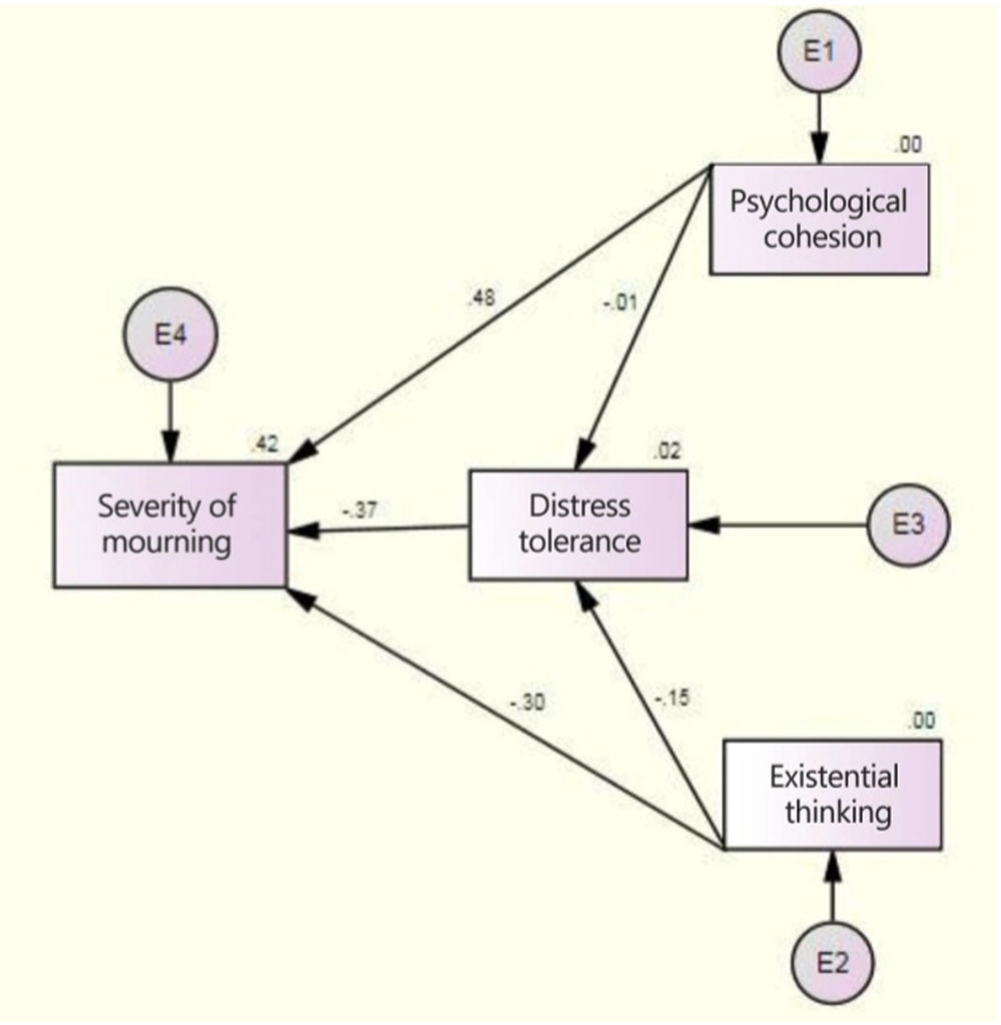
The model for predicting the severity of mourning in the (significant numbers). Figure 2 shows the direct and indirect paths in the significance of numbers.

According to Table 7, the outcomes derived from the bootstrap test indicated that the variable of distress tolerance does not function as a mediator in the relationship between sense of coherence and the severity of mourning experienced by the families of individuals who passed away as a result of Covid‐19. Nevertheless, the distress tolerance variable does play a mediating (indirect) role in the relationship between existential thinking and the severity of mourning experienced by the bereaved families affected by the pandemic (Figures 1 and 2).

**Table 7:** The results of the bootstrap test for the paths of distress tolerance in the relationship between sense of coherence, existential thinking, and the severity of mourning.

Independent variable	Mediator	Dependent variable	Bootstrap	Bias	Standard error	95% confidence interval		Sig
						Upper	Lower	
A sense of coherence	Distress tolerance	Severity of mourning	−0.0139	−0.0008	0.0119	0.0089	−0.0395	0.05
Existential thinking	Distress tolerance	Severity of mourning	0.0602	0.0021	0.0291	0.1239	0.0112	0.03

## 4 DISCUSSION

The primary objective of this investigation was to explore the potential mediating role of distress tolerance in the relationship between existential thinking, sense of coherence, and the severity of mourning in the families of individuals who passed away as a result of COVID‐19. The findings indicated that only the indirect influence of existential thinking on the severity of mourning through distress tolerance was significant, while no direct effect of sense of coherence was found. The results further showed that both existential thinking and sense of coherence, as well as distress tolerance, had a direct and statistically significant impact on the severity of mourning. Furthermore, the direct impact of existential thinking on distress tolerance was also found to be significant.

The research conducted by Bolen and Connor^9^ investigated the correlation between sense of coherence and the severity of mourning experienced by families who lost their loved ones due to COVID‐19. The study found that attributing meaning to the events surrounding the loss can have a positive impact on bereaved individuals, which is consistent with the current research's findings regarding the relationship and direct and significant influence of sense of coherence on the severity of mourning.

Antonovsky posited that individuals with a high level of sense of coherence are less likely to experience stress and anxiety during challenging situations, and they are also less prone to disappointment and depression when facing difficult circumstances.^21^ Consequently, sense of coherence is considered a vital factor in promoting health and positive outcomes, and it is an effective means of coping with problems.^22^ In this regard, having a sense of coherence enables the families of the deceased to perceive life's internal and external stressors in an orderly, predictable, and understandable way.

The present study is in accordance with previous research that examines the direct and indirect impacts of existential thinking on the severity of mourning experienced by families of those who have passed away from Covid‐19. Studies (Brassai et al.,^13^ Dargahi et al.,^14^ and Mason et al.)^15^ have demonstrated that an existential mindset can promote beneficial behaviors, such as improving distress tolerance, through individuals' search for the meaning of life. Moreover, research has suggested that existential thinking can heighten nurses' empathy toward Covid‐19 patients by reducing health anxiety.^14^

How individuals perceive the loss of their loved ones following their passing is influenced by various factors. Individuals who engage in deep reflection are likely to exhibit greater acceptance and flexibility in coping with the loss. By confronting existential issues, individuals can develop a potential capacity for personal growth and increased awareness of the struggles associated with existence.^23^ This heightened awareness can lead to an increase in distress tolerance, ultimately resulting in a reduction in the severity of mourning experienced by the bereaved family members. Some studies have suggested that mediating factors, such as the meaning of life, coping strategies, and religiosity, may play a role in the relationship between existential thinking and grief‐related problems.^24^ Giving meaning to life is a category of existential thinking that is dependent on an individual's attitude toward life.^25^ According to the conducted research, ascribing meaning to problems and suffering is a fundamental aspect of living purposefully, adapting to stress, and enduring distress.^26^ Research investigating the relationship between meaning in life and psychopathology has demonstrated that a low level of meaning in life is associated with various mental health issues, such as addiction disorders, depression, hopelessness, and suicide.^27^ Given the close link between existential thinking and the meaning of life, individuals who perceive life to be more meaningful are better equipped to face life's challenges, process new information, and maintain a broader and more positive outlook for their future. As such, having a sense of meaning in life and engaging in existential thinking can serve as beneficial coping mechanisms, enabling individuals to better endure difficult times, including the mourning period following the loss of loved ones.

## 5 CONCLUSION

The present study's findings, which align with previous research, demonstrate that existential thinking can impact the severity of mourning experienced by families who have lost loved ones to Covid‐19 by promoting distress tolerance. Therefore, emphasizing an existential perspective and ascribing significance to life as a psychological safeguard can assist bereaved individuals in processing and accepting their grief.

## AUTHOR CONTRIBUTIONS

**Soheila Khalafi kasalani**: Data curation; formal analysis. **Mina Karami**: Data curation; investigation; resources. **Shahriar Dargahi**: Conceptualization.

## CONFLICT OF INTEREST STATEMENT

The authors declare no conflict of interest.

## ETHICS STATEMENT

The present research is derived from a research project with the financial support of Islamic Azad University, Ardabil branch and has code of ethics number IR.IAU.ARDAB.ILREC.1401.055. Also, the authors claim that this study complies with the 1975 Declaration of Helsinki, as revised in 2008 (informed consent).

## TRANSPARENCY STATEMENT

The lead author Shahriar Dargahi affirms that this manuscript is an honest, accurate, and transparent account of the study being reported; that no important aspects of the study have been omitted; and that any discrepancies from the study as planned (and, if relevant, registered) have been explained.

## Data Availability

Data is available on request from the authors. The data that support the findings of this study are available from the corresponding author upon reasonable request.

## References

[1] Menichetti Delor JP , Borghi L , Cao di San Marco E , Fossati I , Vegni E . Phone follow up to families of COVID‐19 patients who died at the hospital: families' grief reactions and clinical psychologists' roles. Int J Psychol. 2021;56(4):498‐511. 33511652 10.1002/ijop.12742 PMC8013378

[2] Wetherell JL . Complicated grief therapy as a new treatment approach. Dialogues Clin Neurosci. 2012;14(2):159‐166. 22754288 10.31887/DCNS.2012.14.2/jwetherell PMC3384444

[3] Laranjeira C , Moura D , Salci MA , et al. A scoping review of interventions for family bereavement care during the COVID‐19 pandemic. Behav Sci. 2022;12(5):155. 35621452 10.3390/bs12050155 PMC9137947

[4] O'Cleirigh C , Ironson G , Smits JAJ . Does distress tolerance moderate the impact of major life events on psychosocial variables and behaviors important in the management of HIV? Behav Ther. 2007;38(3):314‐323. 17697855 10.1016/j.beth.2006.11.001 PMC2567911

[5] Simons JS , Gaher RM . The distress tolerance scale: development and validation of a self‐report measure. Motiv Emot. 2005;29(9):83‐102.

[6] McHugh RK , Kertz SJ , Weiss RB , Baskin‐Sommers AR , Hearon BA , Björgvinsson T . Changes in distress intolerance and treatment outcome in a partial hospital setting. Behav Ther. 2014;45(2):232‐240. 24491198 10.1016/j.beth.2013.11.002 PMC4191891

[7] Choi H , Cho C , Lee H . Complicated grief, PTSD, and PTG in bereaved family: moderating effect of resilience and family support. J Loss Trauma. 2023;28(2):145‐160.

[8] Kaya‐Demir D , Çırakoğlu OC . The role of sense of coherence and emotion regulation difficulties in the relationship between early maladaptive schemas and grief. Death Stud. 2022;46(6):1372‐1380. 34159890 10.1080/07481187.2021.1936295

[9] Boelen PA , O'connor M . Is a sense of coherence associated with prolonged grief, depression, and satisfaction with life after bereavement? A longitudinal study. Clin Psychol Psychother. 2022;29(5):1599‐1610. 35912828 10.1002/cpp.2774 PMC9804467

[10] Yalnizca‐Yildirim S , Cenkseven‐Önder F . Sense of coherence and subjective well‐being: the mediating role of hope for college students in Turkey. Curr Psychol. 2023;42(15):13061‐13072.

[11] Fasanghari M , Roshan Chesli R , Allame Z , Ertezaee B . Examination of the psychometric properties of the persion version of the scale for existential thinking. Clin Psychol Personality. 2020;18(2):173‐182.

[12] Asgari P , Bozorgi ZD . The effectiveness of healthy lifestyle training and existential therapy on distress tolerance, health concerns and blood pressure in elderly people with hypertension. Curr Psychol. 2023;42(16):13951‐139519.

[13] Brassai L , Piko BF , Steger MF . Existential attitudes and Eastern European adolescents' problem and health behaviors: highlighting the role of the search for meaning in life. Psychol Rec. 2012;62:719‐734.

[14] Dargahi S , Ayadi N , Kiani A , Ahmadboukani S . The moderating role of existential thinking in the association between health anxiety and nurse empathy with Covid‐19 patients. J Occup Health Epidemiol. 2022;11(2):114‐120.

[15] Mason HD . The relationship between existential attitudes and professional quality of life among nursing students. J Psychol Africa. 2018;28(3):233‐236.

[16] Shams J , Azizi A , Mirzaei A . Correlation between distress tolerance and emotional regulation with students smoking dependence. Hakim Res J. 2010;13(1):8‐11.

[17] Allan BA , Shearer B . The scale for existential thinking. Int J Transp Studies. 2012;31(1):21‐37.

[18] Barrett TW , Scott TB . Development of the grief experience questionnaire. Suicide Life‐Threatening Behav. 1989;19:201‐215. 10.1111/j.1943-278x.1989.tb01033.x 2749862

[19] Antonovsky A . The structure and properties of the sense of coherence scale. Soc Sci Med. 1993;36(6):725‐733. 8480217 10.1016/0277-9536(93)90033-z

[20] Mahammadzadeh A , Poursharifi H , Alipour A . Validation of sense of coherence (SOC) 13‐item scale in Iranian sample. Procedia‐Soc Behav Sci. 2010;5:1451‐1455.

[21] Valtonen M , Raiskila T , Veijola J , et al. Enhancing sense of coherence via early intervention among depressed occupational health care clients. Nord J Psychi. 2015;1:1‐9. 10.3109/08039488.2015.1011230 25739527

[22] Gómez‐Salgado J , Domínguez‐Salas S , Romero‐Martín M , Ortega‐Moreno M , García‐Iglesias JJ , Ruiz‐Frutos C . Sense of coherence and psychological distress among healthcare workers during the COVID‐19 pandemic in Spain. Sustainability. 2020;12(17):6855.

[23] Giannone DA , Kaplin D , Francis LJ . Exploring two approaches to an existential function of religiosity in mental health. Mental Health, Religi Culture. 2019;22(1):56‐72.

[24] Routledge C , Juhl J . When death thoughts lead to death fears: mortality salience increases death anxiety for individuals who lack meaning in life. Cogn Emot. 2010;24(5):848‐854.

[25] Korte J , Cappeliez P , Bohlmeijer ET , Westerhof GJ . Meaning in life and mastery mediate the relationship of negative reminiscence with psychological distress among older adults with mild to moderate depressive symptoms. Eur J Ageing. 2012; 9(4):343‐351. 28804433 10.1007/s10433-012-0239-3 PMC5549108

[26] Lau CL , Feher A , Wilson CA , Babcock SE , Saklofske DH . Resiliency, meaning in life, and life satisfaction: an examination of moderating effects. Acción Psicológica. 2018;15(2):5‐14.

[27] Marco JH , Pérez S , García‐Alandete J , Moliner R Meaning in life in people with borderline personality disorder. Clin Psychol Psychother 2017;24(1):162‐170. 26639791 10.1002/cpp.1991

